# Association between Periodontal Diseases and Polycystic Ovary Syndrome: A Systematic Review

**DOI:** 10.3390/jcm9051586

**Published:** 2020-05-23

**Authors:** Cecilia Fabiana Márquez-Arrico, Javier Silvestre-Rangil, Laura Gutiérrez-Castillo, Mayte Martinez-Herrera, Francisco Javier Silvestre, Milagros Rocha

**Affiliations:** 1 Department of Stomatology, University Hospital Doctor Peset-FISABIO, 46017 Valencia, Spain; cecilia.fabiana.m@gmail.com (C.F.M.-A.); laugcs95@gmail.com (L.G.-C.); 2 Department of Stomatology, University of Valencia, 46010 Valencia, Spain; silranja@uv.es (J.S.-R.); maytemartinez05@gmail.com (M.M.-H.); 3 Department of Endocrinology and Nutrition, University Hospital Doctor Peset-FISABIO, 46017 Valencia, Spain; 4 CIBER CB06/04/0071 Research Group, CIBER Hepatic and Digestive Diseases, University of Valencia, 46010 Valencia, Spain

**Keywords:** periodontal diseases, chronic periodontitis, polycystic ovary syndrome, insulin resistance

## Abstract

Background: A convergent association between polycystic ovary syndrome (PCOS) and periodontal disease, in particular chronic periodontitis (CP), has recently been proposed. The underlying molecular mechanisms of this association are not fully understood, though it is thought that chronic inflammation is responsible. Therefore, the aim of this study was to evaluate the association between periodontal disease—gingivitis and CP—and PCOS. Materials and Methods: The PICO (Participants, Intervention, Control, and Outcomes) question was as follows: “Is there an association between PCOS and CP?” A systematic review of three databases—PubMed, Embase and Scopus—was performed following PRISMA (Preferred Reporting Items for Systematic Reviews and Meta-Analyses) guidelines. Original studies in human cohorts carried out in the last 10 years and including a control group were eligible for inclusion. Letters to the editor, case reports, and reviews were not considered. Results: Ten articles met all the selection criteria and provided a positive answer to the PICO question. Our review of these articles revealed an association between CP and PCOS, since periodontal parameters were altered more frequently in patients with these conditions than in healthy young women. This altered periodontal response in PCOS was associated with a proinflammatory status that seemed to increase susceptibility to periodontal disease. Conclusion: Patients with PCOS appear to be more susceptible to developing periodontal diseases than women without the pathology.

## 1. Introduction

Periodontal diseases are infectious diseases caused by bacteria that affect the periodontium, which is composed of the gum, periodontal ligament, cement, and alveolar bone. There are two major types of periodontal diseases: gingivitis (gum inflammation) and periodontitis (irreversible destruction of periodontal tissues) [1]. Chronic periodontitis (CP) is initiated by an accumulation of bacterial plaque containing periodontopathic germs, which require the presence of a susceptible host [2]. The presence of supra- and subgingival biofilms triggers the activation of the host’s immune system, primarily with protective objectives, but this ultimately leads to the destruction of periodontal tissues due to the synthesis and release of cytokines, proinflammatory mediators, and matrix metalloproteinases (MMPs) [3], which favour the chronification of low-grade inflammation [4,5]. The high prevalence of periodontal disease in adolescents, adults, and older individuals (it affects 25.4% of adults) makes it a serious public health concern. CP has a significant impact on oral health-related quality of life, especially when the disease worsens and has more detrimental consequences. CP results in a loss of connective tissue and bone support, and is a major cause of tooth loss in adults. It also causes dental mobility, malocclusions, and cysts, and it can cause blood infections such as bacteremia or septicemia [6]. In addition, due to its chronic inflammatory nature, CP is associated with a systemic state of oxidative stress, mitochondrial dysfunction [6], and multiple systemic diseases [7,8]. Diabetes mellitus is a traditional risk factor for CP and a bidirectional association between the two diseases has been established [9]. In recent years, newly discovered interactions between CP and other systemic disorders have been the subject of translational research that has confirmed an association of CP with other insulin-resistance (IR) diseases, such as polycystic ovary syndrome (PCOS), rheumatoid arthritis, and cardiovascular disease (CVD), and with a risk of premature births and even some types of cancer [10,11]. 

PCOS is a complex endocrine disorder characterized by the presence of anovulation, menstrual dysfunction, infertility, and hirsutism. In its typical form, it is frequently associated with obesity (predominantly of the abdominal phenotype), dyslipidemia, IR, and hyperinsulinemia, thereby increasing the risk of type 2 diabetes and CVD [12]. The pathogenesis of PCOS is poorly understood, but chronic infections like those that characterise this disease are associated with an increase in oxidative stress and systemic inflammation, and in lipid peroxidation markers, myeloperoxidase, c-reactive protein (CRP), inflammatory cytokines, adhesion molecules, and blood lymphocytes and monocytes [13,14,15]. These are in turn involved in the higher risk of type 2 diabetes and CVD among PCOS patients, and contribute to IR and hyperinsulinemia [16]. 

Recently, a significant association has been proposed between CP and PCOS. The stimulation and chronic secretion of proinflammatory cytokines associated with periodontal infection contributes to IR. This pathognomonic state of systemic inflammation and IR, present in both CP and PCOS, could be a etiologic mechanism linking these two diseases [17]. 

There are very few published studies on the association between PCOS and CP. Özçaka et al. determined that PCOS can influence gingival inflammation [18]. Porwal et al. observed that women with PCOS had higher rates of gingival inflammation and periodontal destruction than those without the syndrome, confirming a higher prevalence and probability of CP among the former [16]. Based on the available evidence, we aimed to evaluate the association between periodontal disease and PCOS, focusing particularly on CP and the molecular mechanisms (mainly inflammatory) that might be involved. To do this, we have carried out a systematic review.

## 2. Material and Methods

### 2.1. PICO Question

Based on the PRISMA [19] guide (Preferred Reporting Items for Systematic Reviews and Meta-Analyses), we used an evidence-based model for framing a PICO question model (PICO: Participants, Intervention, Control, and Outcomes).

The question posed was the following: Is there an association between PCOS and CP? (P) Participants: women of reproductive age who had undergone medical and dental evaluations. (I) Interventions: evaluation of periodontal status in patients diagnosed with PCOS. (C) Control: healthy women, of similar age and weight, who have undergone medical and dental evaluations. (O) Outcome measures: periodontal clinical parameters and inflammatory and microbiological mediators in patients with and without PCOS.

The following keywords were used in three databases, Pubmed (National Library of Medicine, Washington, DC, USA), EMBASE, and Scopus (Elsevier B.V): “Polycystic ovary syndrome” AND “Periodontitis”.

### 2.2. Selection of Articles

The criteria for the inclusion of articles were: original articles reporting prospective or retrospective case–control or cohort studies carried out in humans in the previous 10 years in which a control group had been included.

**Figure 1:**
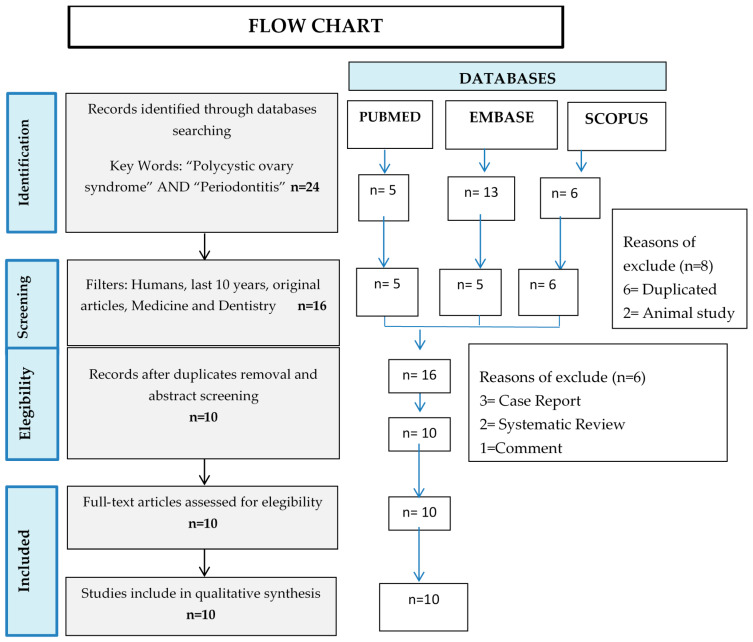
Flow chart of the selection of articles for the systematic review according to PRISMA (Preferred Reporting Items for Systematic Reviews and Meta-Analyses) guidelines.

The following exclusion criteria were established: full text not available, letters to the editor, and case reports and bibliographic reviews. Once the articles had been identified, we screened them by reading the abstracts and excluding the articles whose content did not involve periodontal disease and its relationship with PCOS. Duplicated articles were also eliminated. In this way, we were left only with the articles that met the criteria to answer the PICO question (Figure 1).

### 2.3. Quality of Articles

To perform a critical reading of the studies that met all the selection criteria, the Critical Appraisal Skills Program (CASP) was used [20,21,22]. CASP consists of a checklist of 11 items organised in the following three sections: (A) Are the results of the study valid? (B) What are the results? and (C) Can these results help us in our environment/area? Each question that could be answered affirmatively added a point to the quality score allotted to the article. The maximum score was 11 points (Table 1 and Table 2).

## 3. Results

Twenty-four articles were identified when the keywords were applied to the three databases: five in PubMed, 13 in Embase, and six in Scopus. We then applied the following filters: human studies; published in the last 10 years; full text available; and studies related to medicine and/or odontology. We removed six duplicated articles and two animal studies, thus rendering a new total of 16 articles. After reading the abstracts, we excluded three case reports, two systematic reviews, and one comment to the editor. Ten of these articles met all the selection criteria and answered the PICO question affirmatively. (Figure 1., flow chart of the systematic review according to PRISMA guidelines). Of these ten papers, nine were case–control studies [16,18,23,24,25,26,27,28,29] and one was a randomised clinical trial [30]. Table 3 shows the general characteristics of each of the articles. Table 1 shows the scores of the case–control studies, which reached a maximum score of 9/11. The scores represent the following items: study issues are clearly focused; cases are recruited in an acceptable way; exposure is accurately measured; outcome is accurately measured; confounding factors are addressed; and results are clear and precise. When a critical reading was performed of the randomised clinical trial [30], a score of 9/11 points was obtained. The study issue was clearly focused, the assignment of patients to treatments was randomised, all the patients recruited for the trial were accounted for in the conclusion, the groups were similar at the beginning of the trial, the experimental and intervention group were treated equally, and the benefits were worth the harms and costs (Table 1 and Table 2).

The design of the case and control study was that most often used to study periodontal disease and its relationship with PCOS. The sample size was 52 to 196 participants, and the average age of the subjects studied ranged from 21.0 to 28.6 years old (Table 3).

### 3.1. PCOS Diagnostic Criteria

In all studies, the medical clinical history of the patients was reviewed [16,23,24,25,26,27,28,29,30] and PCOS was diagnosed using the Rotterdam criteria of 2003 and the presence of at least one of the following: (A) polycystic ovaries (presence of >12 follicles measuring 2–9 mm diameter in each ovary and/or an increase in ovary volume > 10 mL); (B) oligo-ovulation and/or anovulation; and (C) hyperandrogenism (clinical and/or biochemical) [31]. One study [30], employed the 2006 criteria of the “Androgen Excess Society” [32], which considers hyperandrogenism (clinical and/or biochemical) as the key feature of PCOS, together with ovarian dysfunction (oligo-anovulation and/or polycystic ovaries) (Table 3). 

### 3.2. Periodontal Diagnostic Criteria

The parameters evaluated in the different studies were measured with a periodontal probe calibrated in millimetres. The following measures were used during periodontal examination in all the articles: presence of dental plaque index (PI); average probing depth of the periodontal pockets (PD); and bleeding on probing (BOP) [16,23,24,25,26,27,28,29,30]. Clinical attachment level (CAL) was also measured in four studies [23,27,29,30] and three studies calculated the gingival index (GI) [23,29,30]. Only one study compared loss of teeth between PCOS women and controls (27] (Table 3). 

### 3.3. Confounding Variables

Most of the studies considered the following confounding variables: obesity (BMI > 30 kg/m^2^), hyperandrogenism, high blood pressure (BP), CVD, diabetes, hyperprolactinemia, congenital adrenal hyperplasia, thyroid disorder, Cushing´s syndrome, hepatic or renal dysfunction, oral contraceptives, steroid hormones, and insulin-sensitizing medications. The presence of nephrotic syndrome, chronic renal failure, or periapical pathology was considered a confounding variable only in the clinical trial [30]. Since tobacco and alcohol are risk factors for periodontal disease, a smoking habit was taken into account in five of the studies [16,23,24,27,30] and drug and alcohol dependence was registered as part of the patient’s medical history in four studies. Only two studies included the presence of cancer as a confounding variable [16,30] (Table 3), while pregnancy and osteoporosis were included in one case–control study [27]. 

Previous periodontal treatment is also relevant when considering the association between periodontal status and PCOS. In this regard, Saglam et al. and Deepti et al. [29,30] included periodontal treatment within the 6 months prior to the study as a confounding variable, while another two studies recorded whether patients and controls had received previous periodontal treatment at any time in their lives [16,27] (Table 3). 

### 3.4. Inflammatory and Oxidative Stress Parameters 

Tumour necrosis factor α (TNFα) and interleukin (IL)-6 represent proinflammatory cytokines that modulate the inflammatory response during periodontal disease. Elevated levels—both in saliva and serum samples—of IL-6 and TNFα in females with PCOS [24] may be involved in IL-17 cytokine expression, which is thought to play a role in the pathogenesis of the disease. In this sense, a significant difference in IL-17A levels was also reported between PCOS subjects with or without gingivitis in serum, gingival crevicular fluid (GCF), and saliva [18]. The proinflammatory members of the IL-17 family may also contribute to the complexity of the inflammatory response in PCOS by influencing gingival inflammation [18]. Higher levels of high sensitivity C-reactive protein (hsCRP) have been observed in serum and blood samples of PCOS patients with periodontal disease compared to periodically healthy PCOS patients [16,30]. Proinflammatory cytokines mediate the host response by inducing and activating the proteolitic function of neutrophils [33]. Activated neutrophils stimulate the production of MMP-9 and myeloperoxidase (MPO), which is considered a potent pro-oxidant, and, consequently, an indicator of oxidative stress. Previous studies have shown increased MPO levels in the GCF of patients with PCOS compared with healthy young women [23], while women with both PCOS and gingivitis were reported to exhibit significantly higher serum levels of MPO and MMP-9 than otherwise systematically healthy women with gingivitis [28]. Other oxidative stress markers have also been the focus of research. Saglam et al. reported higher serum levels of 8-hydroxy-2´-deoxyguanosine (8-OHdG) and malondialdehyde (MDA) in female PCOS patients with or without CP compared to controls [29]. In terms of MMP-8, levels were found to be significantly higher in women with PCOS and gingivitis than in systemically healthy women with gingivitis. Significantly elevated MMP-8 levels in serum were also reported in periodontally healthy women with PCOS in relation to systemically healthy women with gingivitis [26].

### 3.5. Periodontal Microbiota 

High levels of *Porphyromonas gingivalis* and *Fusobacteriumnucleatum* were found in saliva samples, and antibodies for *P. intermedia*, *P. gingivalis*, and *Streptococcus. oralis* were found in serum samples. Accordingly, *Prevotella. intermedia* levels were also significantly higher in women with PCOS and gingivitis compared to systemically or periodontally healthy women [25] (Table 2).

### 3.6. Periodontal Parameters

In all the selected articles, dental check-ups revealed a significantly higher value of PD and BOP in PCOS subjects than in controls [16,18,23,24,25,26,27,28,29,30]. At the same time, there were statistically significant differences in PI values between the two groups, with higher determinations being recorded in PCOS women. The CAL variable had been measured in five studies [16,23,27,29,30], in which GI was higher among PCOS subjects in three [23,29,30] (Table 4).

## 4. Discussion

In the present systematic review, we have confirmed a positive association between periodontal pathologies and PCOS, particularly gingivitis and CP. Periodontal examinations revealed that PD, BOP, and PI were significantly higher in women with PCOS versus controls. In addition, CP was diagnosed by determining pathological CAL. This altered periodontal response in PCOS subjects was associated with a proinflammatory status that may have favoured the susceptibility of these patients to periodontal disease. These three parameters (PD, BOP, and PI) are markers of damage in periodontal tissue, though to confirm a diagnosis of periodontitis the presence of gingivitis with pseudopockets (false pockets) should be ruled out, as they can influence PD, PI, and BOP determinations. In this sense, several authors have introduced the CAL variable into their periodontal evaluation, since it measures insertion loss and thus represents the irreversibility of the process. In light of these data, patients with PCOS would appear to be more susceptible to developing periodontitis and other periodontal diseases, such as gingivitis, than women who do not suffer the pathology.

The studies in this review employed fairly clear criteria to assess the periodontal status of patients. At the same time, the diagnosis of PCOS was based on the Rotterdam criteria (a group of PCOS consensus workshops sponsored by ESHRE/ASRM, 2004) [31] and the “Ferriman Gallwey” escalation [18]. The fact that there was uniformity among the criteria for diagnosing patients made it easier to compare the results of the different studies. All PCOS patients were newly diagnosed and of a similar age (from 21.0–28.6 years old). All the articles evaluated clinical and immunoinflammatory mediators in PCOS and healthy women, though only one considered the microbiological profile of saliva and serum antibodies [25]. 

The results of the present systematic review confirm that PCOS patients are more vulnerable to develop periodontal diseases such as gingivitis and periodontitis. Dursun et al. were the first to report an association between periodontal disease and PCOS [23]; however, since then, only a limited number of studies have further explored this association. Periodontal diseases are infectious, and therefore trigger a local inflammatory response that is maintained over time in the case of periodontitis. Özçaka et al. determined that PCOS could influence gingival inflammation by altering IL-17 [18]. In PCOS patients, this local inflammation is further exacerbated by systemic inflammation. Among other factors, alteration of the lipid profile, which is characteristic of PCOS patients, triggers the release of proinflammatory cytokines such as TNFα, thereby contributing to the pathogenesis of periodontal disease [34,35]. In this sense, Özçaka et al. reported an increased inflammatory response in PCOS patients with gingivitis compared to healthy patients with gingivitis, detecting lower levels of IL-6 in saliva, serum, and GCF, and an increase in salivary TNFα levels in the former group [24]. Porwal et al. observed that women with PCOS displayed higher measures of periodontal inflammation and breakdown than women without PCOS, thus demonstrating an increased prevalence and likelihood of suffering periodontitis in the former [16]. It is thought that a high level of hsCRP in the blood represents an increased risk of heart attack [36]. In PCOS patients with periodontal disease, an increase in systemic hsCRP levels has been observed [16,30], pointing to a synergistic etiologic factor which may be mediated periodontally.

These responses are associated with the induction of other mediators such as MPO and neutrophil elastase [28]; both synthesised by neutrophils, which play a central role in the initial host inflammatory response against periodontal pathogens. These mediators promote and intensify local and systemic inflammation, the stimulation of MMPs, loss of attachment, and bone resorption [26,28]. Furthermore, chronic periodontitis in patients with PCOS has been associated with an increase in oxidative stress markers in serum and saliva, and a decrease in total antioxidant status levels in serum, which contribute to increased oxidative stress [29].

In 2014, Akcali et al. carried out the first study to investigate the association between oral microbiota in saliva and serum antibody responses and gingival inflammation in PCOS [25]. They reported some differences in microbiological components between PCOS patients and controls with periodontal disease. PCOS patients with gingivitis displayed higher levels of *P. gingivalis* and *F. nucleatum* in saliva samples, and antibodies for *P. intermedia*, *P. gingivalis*, and *S. oralis* in serum [25]. As a systemic endocrine condition, PCOS may quantitatively affect the composition of oral microbiota and thus attribute to the enhanced systemic response to selective members of this microbial community.

## 5. Conclusions

To summarise, the results of the present systematic review support an association between periodontal disease and PCOS, as we confirm that periodontal parameters are altered more frequently in PCOS patients than in healthy young women, especially when gingivitis and CP are present. This response could be initially mediated by a local and systemic proinflammatory environment—a pathophysiological mechanism common to both diseases—that favours a pro-oxidant status, thus leading to oxidative stress and eventually to an irreversible destruction of periodontal tissue. We have previously established an association between oxidative stress markers and PCOS [13] and CP [37] in independent studies; however, it is unclear how these two pathologies interact synergistically to exacerbate the burden on cellular pathways. In this context, additional research is warranted in order to clarify the relationship between PCOS and periodontal diseases. 

## Figures and Tables

**Table 1:** CASP quality assessment of the reviewed case–control papers [19].

Authors, Year	Section A: Are the Results of the Trial Valid?						Section B: What are the Results?			Section C: Will the Results Help Locally?		
	Item 1	Item 2	Item 3	Item 4	Item 5	Item 6	Item 7	Item 8	Item 9	Item 10	Item 11	Total Quality Score (0–11)
Akcali A., 2014 [25]	Yes	Yes	Yes	Yes	Yes	No	Yes	Yes	Yes	No	Yes	9
Akcali A., 2015 [26]	Yes	Yes	Yes	Yes	Yes	No	Yes	Yes	Yes	No	Yes	9
Akcali A., 2017 [28]	Yes	Yes	Yes	Yes	Yes	No	Yes	Yes	Yes	No	Yes	9
Dursun E., 2011 [23]	Yes	Yes	Yes	Yes	Yes	No	Yes	Yes	Yes	No	Yes	9
Ozcaka O., 2012 [24]	Yes	Yes	Yes	Yes	Yes	No	Yes	Yes	Yes	No	Yes	9
Ozcaka O., 2013 [18]	Yes	Yes	Yes	Yes	Yes	No	Yes	Yes	Yes	No	Yes	9
Porwall S. 2014 [16]	Yes	Yes	Yes	Yes	Yes	No	Yes	Yes	Yes	No	Yes	9
Rahimnejad M., 2015 [27]	Yes	Yes	Yes	Yes	Yes	No	Yes	Yes	Yes	No	Yes	9
Saglam E., 2017 [29]	Yes	Yes	Yes	Yes	Yes	No	Yes	Yes	Yes	No	Yes	9

Abbreviation: CASP, Critical Appraisal Skills Program. Item 1: Study issue is clearly focused; Item 2: Cohort is recruited in an acceptable way; Item 3: Exposure is accurately measured; Item 4: Outcome is accurately measured; Item 5: Confounding factors are addressed; Item 6: Follow-up is long and complete; Item 7: Results are clear; Item 8: Results are precise; Item 9: Results are credible; Item 10: Results can be applied to the local population; Item 11: Results fit with available evidence.

**Table 2:** CASP quality assessment of the reviewed randomised controlled trial papers [20].

Authors, Year	Section A: Are the Results of the Trial Valid?						Section B: What are the Results?			Section C: Will the Results Help Locally?		
	Item 1	Item 2	Item 3	Item 4	Item 5	Item 6	Item 7	Item 8	Item 9	Item 10	Item 11	Total Quality Score (0–11)
Deepti, 2017 [30]	Yes	Yes	Yes	No	Yes	Yes	Treatment improved statistically (*p* < 0.05) PI, GI, BOP, PD, CAL.	*P* < 0.001 (in PI, GI, BOP, PD and CAL) Confidence Interval not available.	No	Yes	Yes	9

Abbreviation: CASP, Critical Appraisal Skills Program. Item 1: Study issue is clearly focused; Item 2: Was the assignment of patients to treatments randomised?; Item 3: Were all of the patients who entered the trial properly accounted for at its conclusion?; Item 4: Were patients, health workers, and study personnel “blind” to treatment?; Item 5: Were the groups similar at the start of the trial; Item 6: Aside from the experimental intervention, were the groups treated equally?; Item 7: How large was the treatment effect?; Item 8: How precise was the estimate of the treatment effect?; Item 9: Can the results be applied to the local population, or in your context?; Item 10: Were all clinically important outcomes considered?; Item 11: Are the benefits worth the harms and costs?. Periodontal parameters abbreviations: BOP, bleeding on probing; CAL, clinical attachment loss; GI, gingival index; PI, plaque index; PD, probing depth.

**Table 3:** General characteristics of the studies included in the systematic review.

Authors, Year	Study Design	Population	Mean Age in Years	Periodontal Status Diagnostic Methods	PCOS Diagnostic Methods	Confounders Variables Assessed
Akcali A., 2014 [25]	Case–control	N = 125 PCOS-Healthy = 45 PCOS-Gingivitis = 20 Healthy-Controls = 25 Healthy-Gingivitis = 20	25.7	Oral examination, PD, PI, BOP, Work Shop 1999 criteria, saliva and serum samples	Medical History Rotterdam Criteria Ultrasound	BMI > 30 kg/m^2^, hyperandrogenism, high BP, CVD, DM, hyperprolactinemia, congenital adrenal hyperplasia, thyroid disorder, Cushing’s syndrome, hepatic or renal dysfunction, oral contraceptives, steroid hormones, insulin-sensitizing medications
Akcali A., 2015 [26]	Case–control	N = 125 PCOS-Healthy = 45 PCOS-Gingivitis = 20 Healthy-Controls = 25 Healthy-Gingivitis = 20	25.7	Oral examination, PD, PI, BOP, Work Shop 1999 criteria, saliva and serum samples, MMP-8, MMP-I	Medical History Rotterdam Criteria Ultrasound Serum levels	BMI > 30 kg/m^2^, hyperandrogenism, high BP, CVD, DM, hyperprolactinemia, congenital adrenal hyperplasia, thyroid disorder, Cushing´s syndrome, hepatic or renal dysfunction, oral contraceptives, steroid hormones, insulin-sensitizing medications
Akcali A., 2017 [28]	Case–control	N = 125 PCOS-Healthy = 45 PCOS-Gingivitis = 20 Healthy-Controls = 25 Healthy-Gingivitis = 20	25.7	Oral examination, PD, PI, BOP, Work Shop 1999 criteria, saliva and serum samples	Medical History Rotterdam Criteria Ultrasound	BMI > 30 kg/m^2^, hyperandrogenism, high BP, CVD, DM, hyperprolactinemia, congenital adrenal hyperplasia, thyroid disorder, Cushing´s syndrome, hepatic or renal dysfunction, oral contraceptives, steroid hormones, insulin-sensitizing medications
Deepti 2017 [30]	Randomised controlled trail	N = 60 PCOS = 30 Controls = 30	PCOS = 24.0 Controls = 22.6	Oral examination, PI, GI, BOP, PD, CAL	Androgen Excess Society/2006 Criteria	Nephrotic syndrome, chronic renal failure, significant CVD, diabetes mellitus, active cancer, smokers and alcohol dependent, antibiotics, oral contraceptives in the last 3 months, periapical pathology/inflammatory conditions, periodontal treatment within 6 months prior to study
Dursun E., 2011 [23]	Case–control	N = 52 PCOS-Non-obese = 25 Controls = 27	PCOS-non-obese = 22.7 Controls = 24.2	Oral examination, PD, CAL, GI, BOP, PI, Rx, GCF sample, Spectrophotometric, MPO assay	Medical History Rotterdam Criteria Ultrasound Serum levels	BMI > 30 kg/m^2^, hyperprolactinemia, congenital adrenal hyperplasia, thyroid disorder, Cushing´s syndrome, androgen-secreting tumours, smoking, oral contraceptives
Ozcaka O., 2012 [24]	Case–control	N = 73 PCOS-gingivitis = 30 PCOS-healthy = 31 Controls = 12	PCOS-gingivitis = 23.5 PCOS-healthy = 21.0 Controls = 28.5	Oral examination, PD, BOP, PI, saliva sample, GCF sample	Medical History Rotterdam Criteria Ultrasound Serum levels Ferriman Gallwey score	BMI > 30 kg/m^2^, androgen-secreting tumours, congenital adrenal hyperplasia, thyroid disorders, DM, hyperprolactinemia, Cushing´s syndrome, high BP, hepatic and renal dysfunction, oral contraceptives, steroid hormones, insulin-sensitizing drugs, alcohol, smokers
Ozcaka O., 2013 [18]	Case–control	N = 73 PCOS-gingivitis = 30 PCOS-healthy = 31 Controls = 12	PCOS-gingivitis = 23.5 PCOS-healthy = 21.0 Controls = 28.5	Oral examination, PD, BOP, PI, saliva sample, GCF sample,	Medical History Rotterdam Criteria Ultrasound Serum levels Ferriman Gallwey score	BMI > 30 kg/m^2^, hyperandrogenism, thyroid disorders, hyperprolactinemia, CVD, DM, high BP, oral contraceptives, steroid hormone, insulin-sensitizing drugs
Porwal S., 2014 [16]	Case–control	n = 126 PCOS = 41 PCOS-treatment = 45 Healthy controls = 40	PCOS = 23.1 PCOS-treatment = 22.7 Healthy controls = 23.5	Oral examination, PD, PI, BOP, CAL, GI	Medical History Rotterdam Criteria Ultrasound Serum levels WC and WHR hsCRP serum level	BMI > 30 kg/m^2^, thyroid disorders, hyperprolactinemia, androgen-secreting tumours, chronic inflammatory diseases, DM, CVD, cancer, smoking, alcohol, antibiotics, periodontal treatment, aggressive periodontitis
Rahimnejad ME., 2015 [27]	Case–control	n = 196 PCOS = 98 Healthy controls = 98	PCOS = 29.1 Healthy = 28.6	Oral examination, BOP, PD, CAL, PI, tooth loss American Academy of Periodontology Criteria	Medical History Rotterdam Criteria Ultrasound Serum levels	BMI > 25 kg/m^2^, pregnancy, osteoporosis, antibiotics, smoking, periodontal treatment, malignancies
Saglam E., 2017 [29]	Case–control	n = 88 PCOS without CP = 22 PCOS with CP = 22 Healthy with CP = 22 Healthy without CP = 22	PCOS without CP = 27.6 PCOS with CP = 28.6 Healthy with CP = 28.2 Healthy without CP = 27.8	Oral examination, PD, CAL, GI, PI, BOP MDA level 8-OHdG level TAS	Medical History Rotterdam Criteria Ultrasound Serum levels WC and WHR	BMI > 25 kg/m^2^, HbA1c > 6,5%, OGTT-2h > 200 mg/dL, not taken medication within the previous 3 months including antibiotics, oral contraceptives, steroid hormones, hypertensive medications, insulin-sensitizing drugs, periodontal therapy in the previous 6 months, androgen-secreting tumours, congenital adrenal hyperplasia, thyroid disorders, DM, hyperprolactinemia, Cushing´s syndrome

Abbreviations: BMI, body mass index; BOP, bleeding on probing; BP, blood pressure; CAL, clinical attachment loss; CVD, cardiovascular disease; DM, diabetes mellitus, GCF, gingival crevicular fluid; GI, gingival index; MDA, malondialdehyde; MMP, matrix metalloproteinase; MPO, myeloperoxidase; OGTT-2h, 2 h oral glucose tolerance test; 8-OHdG, hidroxi-deoxi-guanosina; PCOS, polycystic ovary syndrome; PI, plaque index; PD, probing depth; TAS, total antioxidant status.

**Table 4:** Primary outcomes of the studies included in the systematic review.

Altered Parameters in Patients with PCOS and Periodontal Disease			
Authors	Clinical	Immunoinflammatory	Microbiological
Akcali A., 2014 [25]	PD, BOP, PI	NA	Saliva: *Porphyromona. gingivalis Fusobacterium nucleatum* Serum antibodies: *Prevotella. intermedia, Porphyromona gingivalis, Streptococcus oralis*
Akcali A., 2015 [26]	PD, BOP, PI	MMP-8/TIMP-1 ratio	NA
Akcali A., 2017 [28]	PD, BOP, PI	Salivary MMP-9 and neutrophil elastase, MMP-9/TIMP-1 ratio Serum MMP-9 and MPO	NA
Deepti 2017 [30]	PD, BOP, PI, CAL, GI	Serum hsCRP	NA
Dursun E. 2011 [23]	PD, GI, BOP, PI	MPO and NO in GCF	NA
Ozcaka O., 2012 [24]	PD, BOP, PI	IL-6 in GCF, saliva and serum, TNFα in saliva	NA
Ozcaka O., 2013 [18]	PD, BOP, PI	IL-17A, IL-F and IL-A/F in serum, IL-17A and IL-17F in GCF and saliva	NA
Porwall S., 2014 [16]	PD, BOP, CAL PCOS newly diagnosed without medical treatment had 2.88 times increased likelihood of having moderate periodontitis.	hsCRP	NA
Rahimnejad ME., 2015 [27]	BOP, PI, CAL	NA	NA
Saglam E., 2017 [29]	PD, BOP, PI, CAL, GI	Serum and salivary 8-OHdG, MDA and TAS levels	NA

Abbreviations: BOP, bleeding on probing; CAL, clinical attachment loss; GCF, gingival crevicular fluid; GI, gingival index; hsCRP, high sensitivity C-reactive protein; IL, interleukin; MDA: malondialdehyde; MMP, matrix metalloproteinase; MPO, myeloperoxidase; NA, not applicable; NO, nitric oxide; 8-OHdG, 8-hydroxy-2´-deoxyguanosine; PCOS, polycystic ovary syndrome; PI, plaque index; PD, probing depth; TAS: total antioxidant status.
